# Neonatal mortality and its predictors among neonates in Jabitehnan district, Northwest Ethiopia: A single‐arm retrospective cohort study

**DOI:** 10.1002/hsr2.1613

**Published:** 2023-10-09

**Authors:** Anteneh Mengist Dessie, Dabere Nigatu, Zemenu Shiferaw Yadita, Yalemwork Anteneh Yimer, Anteneh Kassa Yalew, Eden Workneh Aychew, Sefineh Fenta Feleke

**Affiliations:** ^1^ Department of Public Health, College of Health Science Debre Tabor University Debre Tabor Ethiopia; ^2^ Department of Reproductive Health and population studies, School of public health, College of Medical & Health Sciences Bahir Dar University Bahir Dar Ethiopia; ^3^ Department of pediatric and child health, Tibebe Ghion specialized hospital, college of medicine and health science Bahir Dar university Bahir Dar Ethiopia; ^4^ Department of Public Health, College of Health Science Wolkite University Wolkite Ethiopia; ^5^ Department of Midwifery, College of Health Science Debre Tabor University Debre Tabor Ethiopia; ^6^ Department of Public Health, College of Health Science Woldia University Woldia Ethiopia

**Keywords:** Jabitehnan district, neonatal mortality, Northwest Ethiopia, predictors

## Abstract

**Background and Aims:**

Neonatal period is the most vulnerable time in which children face the greatest risk of death. Worldwide, each year, millions of newborns died in the first month of life. Sub‐Saharan Africa, Ethiopia, in particular, is largely affected. However, there is a dearth of information regarding the survival status of neonates and determinants of their mortality in the study area. Therefore, this study was aimed at investigating neonatal mortality and its predictors in Jabitehnan district, Northwest Ethiopia.

**Method:**

A single‐arm community‐based retrospective cohort study was conducted in March 2021 among 952 neonates born between August 2020 and February 2021. Data were collected by a semi‐structured questionnaire, and a multistage stratified sampling technique was employed to select one urban and 10 rural kebeles from the district. Then, the total sample size was proportionally allocated to these selected kebeles. Neonatal death was ascertained by community diagnosis. Kaplan–Meier curve was used to estimate survival time. Cox regression was used to identify factors, the hazard ratio was estimated, and a *p*‐value < 0.05 was considered statistically significant.

**Results:**

The neonatal mortality rate was 44 (95% confidence interval [CI]: 33–60) per 1000 live births; and the incidence rate was 1.64 (95% CI: 1.21–2.23) per 1000 neonate days. Three‐quarters of deaths occurred in the first week of life. Medium household wealth index (adjusted hazard ratio [AHR] = 3.54; 95 CI: 1.21–10.35), increased number of pregnancies (AHR = 1.22; 95%CI: 1.01–1.47), being male (AHR = 2.45, 95% CI: 1.12–5.35) and not starting breastfeeding in the first hour of life (AHR = 4.00; 95% CI: 1.52–11.10) were found to be predictors of neonatal mortality.

**Conclusion:**

Neonatal mortality was high compared to the national target. Wealth, number of pregnancies, sex of the neonate, and breastfeeding initiation were factors associated with neonatal death. Hence, strengthening interventions such as providing sexual education in the population, considering households with a medium wealth index in the exemption service, and counseling mothers about early breastfeeding initiation would improve neonatal survival.

## 1 INTRODUCTION

Neonatal mortality (NM) refers to the baby deaths that occur throughout the first 28 days after birth (the neonatal period). This period is the most vulnerable time for a child's survival, which holds the highest concentrated risk of death in a human lifetime.^1^ Although being a newborn is not an illness, massive numbers of youngsters die shortly after birth: several of them within the first 4 weeks of life, and most of these throughout the first week.^2^

Neonatal mortality is still a very important public health problem in the world, particularly in developing countries. In 2019, 2.4 million children died in the first month of life globally, 17 deaths per 1000 live births. Sub‐Saharan Africa (SSA) is the region with the highest NM at 27 deaths per a thousand live births.^3^ In 2017, together, south Asia and sub‐Saharan Africa accounted for 79% of the total burden of neonatal deaths.^4^

In the last decade, efforts have been made globally as well as nationally to overcome the neonatal deaths. For instance, it remained an “unfinished agenda” of Millennium Development Goals (MDGs) which was prolonged to Sustainable Development Goals (SDGs).^5^ The Ethiopian government conjointly formulates and implements so many policies including National Newborn and Child Survival Strategy,^6^ Kangaroo Mother Care,^7^ and Health Sector Transformation Plan^8^ to improve childhood survival. However, despite the world has created substantial progress in child survival within the past decades, neonatal death has remained comparatively unchanged, and the percent share of under‐five mortality was increased.^9^ The Ethiopian Demographic and Health Survey reported an NM of 39 per 1000 live births in 2005, 29 per 1000 live births in 2016 and 30 per 1000 live births in 2019. Of the total under‐five deaths in 2019, 54.5% occurred in the first month of life, increasing from 29.5% in 2000.^10^ Also globally, the percent share of NM among all under‐five mortality was up from 40% in 1990 to 47% in 2019.^3^, ^11^

Several studies in Ethiopia showed that the neonatal mortality ranges from 3.5%^12^ to 34.9%.^13^ Studies have also indicated that rural residence, far‐flung physical accessibility of health facilities,^14^, ^15^ male sex of newborns,^14^, ^16^, ^17^, ^18^, ^19^ low parental education level,^12^, ^15^ both young and old maternal age,^12^, ^14^, ^15^, ^20^, ^21^ low economic status,^20^, ^22^, ^23^ both preterm and postterm pregnancy,^12^, ^19^, ^20^, ^24^ low birth weight,^12^, ^14^, ^15^, ^18^, ^20^, ^22^, ^24^, ^25^ higher birth order,^12^, ^16^, ^20^, ^21^ short birth interval,^14^, ^16^, ^18^, ^20^, ^21^ not initiating breastfeeding with 1 h of life,^15^, ^17^, ^25^, ^26^ multiple gestation,^12^, ^18^, ^19^, ^20^ cesarean section delivery,^16^, ^21^ home delivery,^12^, ^20^, ^24^, ^25^ and not attending antenatal and postnatal care^16^, ^17^, ^19^, ^20^, ^25^ were the risk factors for neonatal death.

Despite the highest prevalence of neonatal mortality and stagnant decrement in Ethiopia, majorities of the previous pieces of evidence on neonatal mortality were facility‐based,^15^, ^27^, ^28^ which may not show the actual deaths on the ground^15^, ^27^ and it possibly miss some factors since most of them used a retrospective chart review.^29^ In addition, those women delivered in health facilities had better educational status, good household wealth status, urban residents, history of illness episodes, and access to health facilities.^10^, ^30^ The health facility delivery in Ethiopia, mainly, in the Amhara region, is also at a lower level,^10^ and home delivery is rampant in the region. Hence, this community‐based study was aimed at assessing at assessing the neonatal mortality rate (Incidence) and its predictors in the Jabitehnan district, Northwest Ethiopia.

## 2 METHODS AND MATERIALS

### 2.1 Study design, setting, and period

A single‐arm community‐based retrospective cohort study was conducted from March 10 to 30, 2021, to determine the survival status of neonates and predictors in the Jabitehnan district, Amhara region, Northwest Ethiopia.

### 2.2 Population

All neonates born alive within the last 6 months (from August 10, 2020 to February 10, 2021) before the data collection period in the district were the source population for the study. The study population consists of all neonates born alive within the period in the randomly selected Kebeles (the smallest administrative units of Ethiopia that are accountable to their district). However, the study excludes neonates whose mothers died since there are several factors that can be obtained from the mother only.

### 2.3 Sample size and sampling procedure

The sample size was determined by using a sample size calculation for the survival analysis. Different factors were considered in the sample size calculation but the final maximum sample size was calculated by considering postnatal care follow‐up as the major predictor variables^17^ using STATA version 14. The assumptions include; hazard ratio (HR) (3.07), survival probability among unexposed (0.977), confidence interval (CI) (95%), power of the study (80%), alpha (5%), ratio (1:1), a design effect of 1.5, and 5% nonresponse rate. The final sample size was 952.

A multistage stratified random sampling technique was employed to select the study participants. The study was conducted in the Jabitehnan district, which is composed of 41 Kebeles. The Kebeles were divided into two strata: three urban and 38 rural Kebele's. Then, one urban (Jiga 02) and 10 rural (Yelmdar, Arbayitu, Mircha, Wongie, Shimebed, Hodansh, Atat, Goref, Jimmat, and Abasem) Kebeles were selected by a simple random sampling technique. The total number of the eligible neonates was taken from the registration (family folder) for each selected Kebeles. Then the calculated sample size was proportionally allocated to the randomly selected Kebele's. The proportional allocation was done based on the total number of neonates born alive in the last 6 months within each Kebeles. Eligible neonates were identified from the registration (family folder) and delivery registration book. Finally, each study participant was selected by simple random sampling using the computer generation technique. For those households with two mother–neonate pairs, only one of the mother–neonate pair was taken by lottery method.

### 2.4 Study variables and measurement

The dependent variable of the study is the time to death of neonates. The predictor variables were residence (urban/rural), wealth index, educat**i**on of the mother and the father, occupation of the mother and the father, membership of community‐based health insurance (CBHI), estimated distance from the house to the health facility in kilometers, sex of neonate, gestational age at birth, preceding birth interval, child‐size at birth, colostrum feeding, initiation time of breastfeeding, age of mother, gravidity, antenatal care (ANC) visit, iron‐folic acid utilization, tetanus toxoid (TT) vaccination, postnatal care (PNC) visit, multiple gestation, family planning utilization, mode of delivery, and place of delivery.

**Pre‐lacteal feeding:** giving newborns any solid or liquid foods other than breast milk before breastfeeding initiation or before breast milk comes in, usually in the first few days of life.

**Optimal birth interval:** refers to a minimum of 24 months after a live birth‐to‐pregnancy or 33 months birth‐to‐birth intervals.^31^

**Wealth Index of Household**: the wealth index of the household was determined using principal component analysis (PCA) by considering housing condition, house ownership, household assets, and land ownership. The responses of all variables were recoded as 0 and 1, with the higher value receiving a code of 1 and the smaller value receiving a code of 0. Factor scores were produced using variables having a commonality value of greater than 0.5 in PCA. Finally, three quintiles that divide the household in to three groups (poor, medium, and rich) were constructed using the first principal component. Generally, it was done based Ethiopian Demographic and Health Survey principal component analysis method.^32^, ^33^

**Preterm birth:** via considering the Last Normal Menstruation Period (LNMP); if the neonate is born before 37 weeks of gestational age or LNMP is not known and the mother reports the neonate is born before 9 months of gestation.

**Child size:** the child's size at birth was considered as small if the mothers' report for the child's size at birth was either very small or small. On the other hand, the child's size at birth was considered as normal if the mothers' report for the child's size at birth was average, and as big if she reported as big or very big.

### 2.5 Data collection

Data were collected retrospectively using a pre‐tested semi‐structured questionnaire which was developed by reviewing previous works of literature.^15^, ^17^, ^25^, ^28^ The questionnaire had three parts; sociodemographic, neonatal information, and maternal information and service utilization. The survival data were collected from mothers. The neonatal death was confirmed by the community diagnosis (the mothers declare and mourn the death of their neonates based on the norms and customs of the community). A child who died before 28 days of life was an event of interest, and those who are alive during data collection or died after 28 days of life were considered right‐censored. Sample recruitment was performed in each Kebele from the list of mothers obtained in the health post. One data collector was assigned to each Kebeles. The health extension workers under the local authorities have routine family register (Family folder) for vital events and background information (including addresses) of the mothers for each household under their Kebeles. Child birth, maternal death, neonatal death, infant death, and others are regularly documented by the health Extension workers. Finally, the data collector was in touch with the eligible mothers by the help of the health Extension workers and local authorities. Subjects were considered nonrespondent if they were not willing to answer or were unable to finish the interview. Repeat visit was made for those mothers who were not available during the data collection. Another eligible mother was selected if they were not around during the repeat visit.

### 2.6 Follow‐up period

The sampled neonates who were born alive between September 1, 2020 and March 1, 2021 were followed for 28 days. The date of birth of the neonate was considered as the starting point of the retrospective follow‐up. The date of birth was taken from the family folder recorded by the Health extension workers of each Kebeles, While the date of death was taken from the mothers. The mothers declare and mourn the death of their child based on the norms and cultures of the community. The length of time was measured in days and was taken to be the survival time for those who had experienced the event of interest.

### 2.7 Statistical analysis

Data entry and analysis were done by Epi‐data version‐3.1 and STATA™ version‐14, respectively. Missing data were handled using an appropriate method of imputation. Categorical variables were summarized as numbers and percentages and presented using frequency tables and graphs, whereas continuous variables were summarized as mean/median and standard deviations/interquartile range (IQR) based on the distribution of the data. Kaplan–Meier curve was used to estimate survival time. Cox regression was used to identify predictors of neonatal deaths. Initially, Bivariate Cox regression analysis was done to identify variables with a *p*‐value ≤ 0.2, which are candidates for the multivariable Cox regression analysis. Then, using the multivariable Cox regression analysis, adjusted hazard ratios (AHR) were calculated as a measure of association. Cox‐proportional hazard assumption was checked by using both log‐log plot of survival and proportional‐hazards assumption test based on Schoenfeld residuals. Both of these assumptions were met. Multi‐collinearity was also checked by using variance inflation factor (VIF). The goodness of the model fitness was also checked by using the Cox‐Snell residual test. All the tests were two‐sided, and a *p* < 0.05 was set to determine the statistical significance of the tests.

### 2.8 Ethical approval

A written ethical approval letter was taken from Bahir Dar University Research Ethics Review Committee (Ethical approval number: 0112/2021). Moreover, a support letter was secured from the Jabitehnan district. Each mother was informed about the objective of the study, the confidentiality of their data, and the right to refuse participation, and they gave written consent to participate. To ensure confidentiality, the name of the interviewee was not written on the questionnaire. Respondents were assured that the information provided by them was confidential and used only for research.

## 3 RESULT

### 3.1 Sociodemographic characteristics the study participant

Out of 952 samples approached during the study, 926 of the mothers responded to the questionnaire making a response rate of 97.3%. Of these 926 mothers responded, more than half (477 (51.51%)) of mothers were between the ages of 25 and 35. The mothers' median age was 32 with an interquartile range of 36–27 = 9 years old. Nearly all 904 (97.62%) mothers were followers of Orthodox Christianity, and 900 (97.2%) were married. Regarding educational status, around half of 457 (49.35%) of them cannot read and write. Four hundred seventy‐one (50.86%) of the households were in a poor wealth quintile (Table 1).

**Table 1:** Sociodemographic characteristics of mothers of neonates in the Jabitehnan district, Northwest Ethiopia, March 2021 (*n* = 926).

Characteristics	Categories	Frequency	Percent
Age of the mother	15–24	122	13.2
	25–34	477	51.5
	≥35	327	35.3
Residence	Urban	89	9.6
	Rural	837	90.4
Marital status	Married	900	97.2
	Others*	26	2.8
Occupation of the mother	Farmer	785	84.8
	Government employee	23	2.5
	Merchant	42	4.5
	House wife	67	7.2
	Others**	9	1.0
Occupation of the father	Farmer	776	83.8
	Government employee	37	4.0
	Merchant	90	9.7
	Others***	23	2.5
Community‐based health insurance membership	Yes	621	67.1
	No	305	32.9
Education level of mother	Can't read and write	457	49.4
	Read and write only	159	17.2
	Primary education	167	18.0
	Secondary and above	143	15.4
Education level of father	Can't read and write	393	42.4
	Read and write only	211	22.8
	Primary Education	152	16.4
	Secondary and above	170	18.4
Wealth index	Poor	471	50.86
	Medium	179	19.33
	Rich	276	29.81
Distance from nearest health institution	≤5 km	907	97.9
	>5 km	19	2.1

* Others include (divorced, single, and widowed).

** Others include (student and daily laborer).

*** Others include (Student, daily laborer and private/NGO employee.

### 3.2 Maternal characteristics

Of the total of 926 studied neonates, 676 (73%) of them were born to a mother who had at least one antenatal care visit during pregnancy. Six hundred and seventeen (66.63%) of mothers delivered in the health institution and 749 (80.9%) gave birth by spontaneous vaginal delivery. Seven hundred and sixty‐one (82.2%) mothers were multigravida and 588 (63.5%) mothers were using the family planning method just before the pregnancy of the indexed neonate. The median age of mothers at their first marriage was 18 years with a minimum of 7 years and a maximum of 28 years. Seventy‐two (9.46%) of 761 multigravida mothers had a previous history of neonatal mortality. During the pregnancy of the indexed neonate, only 596 (64.4%) of the mothers received two or more tetanus toxoid vaccines (Table 2).

**Table 2:** Characteristics of mothers of neonates in the Jabitehnan district, Northwest Ethiopia, March 2021 (*n* = 926).

Characteristics categories		Frequency	Percent
Gravidity	Primigravida	165	17.8
	Multigravida	372	40.2
	Grand multigravida	389	42.0
Inter‐birth interval	Not applicable	165	17.8
	<33 months	243	26.2
	>=33 months	518	56.0
FP just before the indexed pregnancy	Yes	588	63.5
	No	338	36.5
Method used (*n* = 588)	Oral	28	4.8
	Injectable	480	81.6
	Implant	74	12.6
	IUCD	6	1.0
Type of pregnancy	Single	910	98.3
	Twin	16	1.7
Antenatal care visit	Have no ANC visit	250	27.0
	1–3 visit	495	53.4
	>=4 visit	181	19.5
TT Vaccination in the index pregnancy	Not vaccinated	262	28.3
	TT one	68	7.3
	TT two and above	596	64.4
Iron folate utilization	Not take iron folate	259	28.0
	Take for less than 90 days	563	60.8
	Take for 90 and above day	104	11.2
Place of birth	Home	309	33.4
	Health institution	617	66.6
Mode of delivery	Spontaneous vaginal delivery	749	80.9
	Assisted vaginal delivery	150	16.2
	Cesarean section	27	2.9
Had postnatal care	Yes	209	22.6
	No	717	77.4

Abbreviations: FP, family planning; ICD, intrauterine contraceptive device; TT, tetanus toxoid.

### 3.3 Neonatal characteristics

Of the total 926 neonates, 469 (50.65%) were female making the female‐to‐male ratio about 1:1. According to the perception of mothers on the size of their neonates, 373 (40.3%) reported large size, and only 53 (5.7%) of mothers give birth before 9 months of gestation. Among 916 neonates who did not die before 1 h of birth, 623 (68.01%) were initiated breastfeeding within 1 h of birth and only 103 (11.2%) were not fed colostrum part of the breast milk. The two most common reasons for not breastfeeding the baby immediately after birth were waiting for delivery of the placenta (25.9%) and lack of counseling (23.2%). One hundred and ninety‐seven (21.3%) of mothers applied something to the umbilical cord of their baby, and majorly 173 (87.8%) butter or any other ointment was applied.

### 3.4 Neonatal survival outcome of the follow‐up

**Figure 1:**
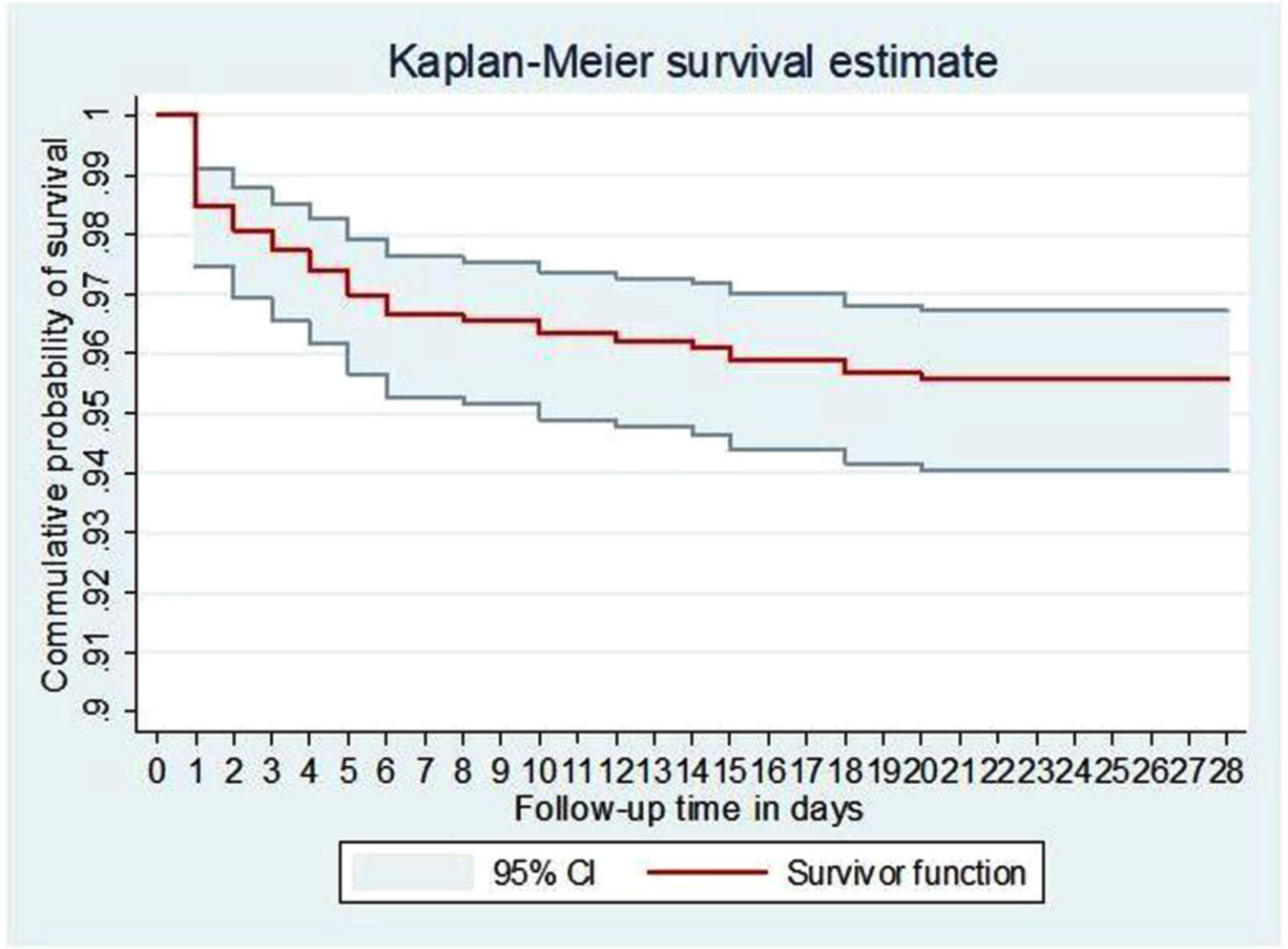
The overall Kaplan‐Meier survival curve with a 95% confidence interval showing the survival time of neonates in Jabitehnan district, Northwest Ethiopia, March 2021.

In this study, the cohort of 926 neonates contributed a total of 25,001 neonate days. The median follow‐up time was 28 days, with an interquartile range of 28 days as well. During the time of follow‐up, 41 deaths were recorded giving the overall neonatal survival 95.6% (95% CI: 94.0–96.7) and making the NMR 44 per 1000 live births. The incidence rate of neonatal mortality was 1.64 (95% CI: 1.21–2.23) per 1000 neonate days of observation (Figure 1). All deaths occurred during the first three consecutive weeks. Three‐quarters, 31 (75.6%) of neonatal deaths occurred in the early phase of the neonatal period especially 14 (34.1%) on the first day of life. The incidence rate of early neonatal mortality was 4.9 (95% CI: 3.4–6.9) per 1000 early neonate days, while late neonatal mortality rate was 0.5 (95% CI: 0.3–1.0) per 1000 late neonate days. The incidence rate of male neonates was 2.22 per 1000 neonate days and 1.10 for female neonates.

### 3.5 Predictors of neonatal mortality

Initially, the bivariate Cox‐regression analysis was done. A total of 14 variables were included in the Bivariate analysis. However, only nine of them showed association with neonatal death at a *p*‐value of ≤ 0.2. These nine variables namely, wealth index, CBHI membership, number of pregnancies, family planning utilization just before the indexed pregnancy, ANC follow‐up, place of birth, neonate's sex, initiation time of breastfeeding, and gestational age at birth were candidates for multivariable regression (Table 3).

**Table 3:** Bivariate Cox proportional hazard regression of factors of neonatal mortality in Jabitehnan district, Northwest Ethiopia, March 2021.

Variables	Categories	Survival status		CHR (95%CI)	*p*‐Value
		Died	Alive		
Wealth index	Poor	20	451	1.69 (0.71–4.00)	0.20
	Medium	14	165	3.17 (1.28–7.85)	0.01
	Rich	7	269	1	
CBHI membership	Yes	23	598	1	
	No	18	287	1.61 (0.87–2.98)	0.13
Number of pregnancy				1.19 (1.05–1.35)	<0.001
FP just before the indexed Px	Yes	20	568	1	
	No	21	317	1.85 (1.00–3.41)	0.04
ANC follow‐up	Yes	26	650	0.64 (0.34–1.20)	0.16
	No	15	235	1	
Place of birth	Home	21	288	2.11 (1.15–3.90)	0.008
	Health facility	20	597	1	
Sex of neonate	Male	27	430	2.00 (1.05–3.82)	0.03
	Female	14	455	1	
Gestational age at birth	Less than 9 months	8	45	4.16 (1.92–9.01)	<0.001
	9 months and above	33	840	1	
Breast feeding initiation	≤1 h	14	614	1	
	>1 h	27	271	4.17 (2.22–7.69)	<0.001
Mode of delivery	Spontaneous vaginal delivery	31	718	1	
	Assisted vaginal delivery	8	142	1.30 (0.6–2.840)	0.49
	Cesarean section	2	25	1.82 (0.43–7.61)	0.41
Postnatal care	Yes	8	201	1	
	No	33	684	1.21 (0.55–2.62)	0.62
TT vaccination in the Index Pregnancy	Not vaccinated	15	247	1	
	TT one	3	65	1.48 (0.77–2.85)	0.22
	TT two and above	23	573	1.12 (0.34–3.78)	0.83
Age of the mother	<35	19	580	1	
	≥35	22	305	2.16 (1.16–3.99)	0.01
Residence	Urban	5	83	1	
	Rural	36	802	0.74 (0.29–1.90)	0.54
Religion of the mother	Orthodox	39	865	1	
	Muslim	2	20	2.13 (0.51–8.83)	0.29

Abbreviations: AC, antenatal care; CBHI, community‐based health insurance; CHR, crude hazard ratio; CI, confidence interval; FP, family planning; Px, pregnancy; TT, tetanus toxoid.

Nine candidate variables were included in the multivariable Cox proportional hazard regression model. However, only wealth index, number of pregnancies, neonate's sex, and initiation time of breastfeeding were significant predictors of neonatal mortality at a *p*‐value < 0.05 (Table 4).

**Table 4:** Multivariable Cox proportional hazard regression of predictors of neonatal mortality in Jabitehnan district, Northwest Ethiopia, March 2021.

Variables	Categories	AOR (95%CI)	*p*‐Value
Wealth index	Poor	1.04 (0.42–2.60)	
	Medium	**3.54** (**1.21–10.35)**	**0.02**
	Rich	1	
CBHI membership	Yes	1	
	No	1.76 (0.84–3.72)	0.13
Number of pregnancy		**1.22** (**1.01–1.47)**	**0.03**
FP just before the indexed Px	Yes	1	
	No	1.15 (0.48–2.74)	0.74
ANC follow‐up	Yes	1.03 (0.43–2.45)	0.09
	No	1	
Place of birth	Home	1.16 (0.44–3.06)	0.76
	Health facility	1	
Sex of neonate	Male	**2.45** (**1.12–5.35)**	**0.02**
	Female	1	
Gestational age at birth	Less than 9 months	1.90 (0.69–5.17)	0.21
	9 months and above	1	
Breast‐feeding initiation	≤1 h	1	
	>1 h	**4.00 (1.52–11.10)**	<**0.001**

Abbreviations: AC, antenatal care; AHR, adjusted hazard ratio; CBHI, community‐based health insurance; CI, confidence interval; FP, family planning; Px, pregnancy.

The hazard of neonatal mortality among neonates who were born to mothers with a medium household wealth index was three (AHR = 3.54; 95% CI: 1.21–10.35) times higher compared to those neonates born from a household with a high wealth index. Every rise in the number of pregnancies was associated with a 22% (AHR = 1.22; 95% CI: 1.01–1.47) the higher hazard of neonatal mortality. Male neonates had a 2.45 (AHR 2.45, 95% CI: 1.12–5.35) times higher hazard of neonatal mortality compared with female neonates. Neonates who did not initiate breastfeeding within 1 h of birth had a 4.00 (AHR = 4.00; 95% CI: 1.52–11.10) times higher hazard of neonatal mortality as compared to those who initiate breastfeeding timely.

## 4 DISCUSSION

The overall aim of this study was to determine the incidence of neonatal mortality and its predictors among neonates in Jabitehnan district, Northwest Ethiopia. Accordingly, the neonatal mortality rate (NMR) was 44 (95% CI: 33–60) per 1000 live births; and the incidence rate of neonatal mortality in this study was 1.64 (95% CI: 1.21–2.23) per 1000 neonate‐days. The medium household wealth index, increased number of pregnancies, being male and breastfeeding initiation within 1 h were found to be predictors of neonatal mortality.

The finding of NMR in this study was in line with the finding from a study conducted in the Jimma zone, Aroresa district, Butajira, and Bangladesh.^14^, ^17^, ^32^, ^33^ However, this rate was higher than studies conducted in India and Pakistan.^34^, ^35^ This might be attributed to the difference in socioeconomic status of the population, and the study area is known to have poor access to basic health services.

On the other hand, the rate of neonatal mortality in this study was lower than similar studies in the hospital setting^28^, ^29^, ^36^, ^37^ and a pooled prevalence report.^38^ This could be partly explained by the fact that this study was conducted at a community level in which the denominator covers many healthy neonates and the resulting rates could be lower. The facts of neonates admitted to the NICU are usually those who need intensive care and held a higher risk of death^26^ will also explain this difference.

This study also revealed that most neonatal deaths occurred in the first 24 h (34.1%) and in the first week of life (75.6%), which is consistent with different studies conducted before^12^, ^15^, ^17^, ^33^ and 2020 WHO report.^3^ This consistent result of major death in the first week of life might be justified as conditions related to labor, intrapartum and immediate newborn care practices were reasons for the majority of neonatal death. It indicates that focus should be given to interventions that are targeted towards intrapartum as well as immediate and early neonatal periods.

Regarding the predictors, the wealth index is one of these factors identified as significant predictors of neonatal mortality. Accordingly, the hazard of neonatal mortality among neonates who were born to mothers with a medium household wealth index was higher as compared to those born from a household with a high wealth index which is supported by other studies.^20^, ^22^ his may be due to differences in the quality of life and access to health services. As the number of pregnancies increases the hazard of neonatal death increase significantly. This is consistent with other studies,^12^, ^20^, ^39^ which possibly due to frequent birth as well as high parity predisposes the newborn to unfavorable health outcomes.

This study also showed that initiation of breastfeeding within the first hour of delivery decreases the hazard of neonatal mortality. Many other studies also confirmed the protective effect of immediate breastfeeding initiation after birth^15^, ^17^, ^25^, ^26^, ^36^ as recommended by WHO. Possibly this is due to colostrum and breast milk containing an ideal nutrient that protects the newborn from a wide variety of infections and promotes their health.

The sex of neonates is found to be one of the important factors that affect neonatal mortality in this study. Male neonates had a higher hazard of neonatal mortality. This is similar to study findings in Ethiopia^14^, ^17^, ^18^, ^19^, ^25^ Bangladesh^32^ Iran,^22^ and the African Great Lakes region.^16^ This could be due to the fact that the neonatal period is the time at which natural differences between males and females were more pronounced. Males are more vulnerable to respiratory and gastrointestinal infections due to high testosterone.

Unexpectedly, in this study, antenatal health care services utilization and place of delivery lack an association with neonatal mortality. Several previous studies reported that lack of antenatal care and giving birth at home was associated with a higher risk of neonatal mortality.^14^, ^16^, ^17^, ^18^, ^20^, ^25^, ^32^ The possible reason might be that most mothers were going to give birth at health institutions when they encountered problems during pregnancy and labor such as PROM, prolonged labor, bleeding.^40^ It makes institutional deliveries are more complicated and more likely to have poor outcomes.

## 5 STRENGTHS AND LIMITATIONS OF THE STUDY

This study was a community‐based study, and it provides the actual figure of the problem in the community. However, a community‐based retrospective study is subjected to recall and social desirability bias, since it relied on women's recall ability. Some mothers may not report the exact date of birth and death of the neonates. To reduce this bias, the data were collected only from neonates born within the last 6 months. It was also impossible to have data on some important clinical variables like hypothermia, jaundice, and birth asphyxia. The study has excluded those neonates whose mother had died, hence, it was prone to selective survival bias. Mother's perception was also used to estimate the size of neonates, which may result in misclassification of birth weight. However, adequate explanation has been given so that mothers can accurately estimate the size of their baby. Finally, since the study is a retrospective cohort, causality may not be established.

## 6 CONCLUSIONS

The study has found that the neonatal mortality in the study area was high as compared with national and global targets. Factors such as wealth status, sex of neonates, number of pregnancies, and initiation time of breastfeeding were found to be predictors of neonatal mortality. Hence, this study suggests, limiting the number of pregnancies through quality family planning service, and preventing early marriage should be emphasized. Counseling on early breastfeeding initiation should be enhanced during their ANC, labor, or postnatal period. Besides, a fee waiver system for basic health services should be recommended to consider the household's wealth status.

## AUTHOR CONTRIBUTIONS

**Anteneh Mengist Dessie**: Conceptualization; data curation; formal analysis; funding acquisition; investigation; methodology; project administration; resources; software; supervision; validation; visualization; writing—original draft; writing—review & editing. **Dabere Nigatu**: Methodology; software; visualization; writing—original draft; writing—review & editing. **Zemenu Shiferaw Yadita**: Conceptualization; project administration; software; supervision; validation; visualization; writing—original draft; writing—review & editing. **Yalemwork Anteneh Yimer**: Project administration; software; visualization; writing—original draft; writing—review & editing. **Anteneh Kassa Yalew**: Conceptualization; formal analysis; methodology; visualization; writing—original draft; writing—review & editing. **Eden Workneh Aychew**: Conceptualization; data curation; formal analysis; investigation; methodology; software; visualization; writing—original draft; writing—review & editing. **Sefineh Fenta Feleke**: Conceptualization; data curation; formal analysis; investigation; methodology; software; visualization; writing—original draft; writing—review & editing.

## CONFLICT OF INTEREST STATEMENT

“Anteneh Mengist Dessie” is an Editorial Board member of Health Science Reports, and an author of this article. To minimize bias, they were excluded from all editorial decision‐making related to the acceptance of this article for publication.

## TRANSPARENCY STATEMENT

The lead author Anteneh Mengist Dessie affirms that this manuscript is an honest, accurate, and transparent account of the study being reported; that no important aspects of the study have been omitted; and that any discrepancies from the study as planned (and, if relevant, registered) have been explained.

## Data Availability

Data are available upon reasonable request.
